# Impact of Market Participation of Indigenous Crops on Household Food Security of Smallholder Farmers of South Africa

**DOI:** 10.3390/su142215194

**Published:** 2022-11-16

**Authors:** Nonkululeko Thandeka Brightness Zondi, Mjabuliseni Simon Cloapas Ngidi, Temitope Oluwaseun Ojo, Simphiwe Innocentia Hlatshwayo

**Affiliations:** 1 African Centre for Food Security, School of Agricultural, Earth and Environmental Sciences, College of Agriculture, Engineering and Science, University of KwaZulu-Natal, Private Bag X01, Scottsville, Pietermaritzburg 3201, South Africa; 2 Centre for Transformative Agricultural and Food Systems, School of Agricultural, Earth and Environmental Sciences, College of Agriculture, Engineering and Science, University of KwaZulu-Natal, Private Bag X01, Scottsville, Pietermaritzburg 3201, South Africa; 3 Department of Agricultural Extension and Rural Resource Management, School of Agricultural, Earth and Environmental Sciences, College of Agriculture, Engineering and Science, University of KwaZulu-Natal, Private Bag X01, Scottsville, Pietermaritzburg 3201, South Africa; 4 Department of Agricultural Economics, Obafemi Awolowo University, Ile-Ife 22005, Nigeria; 5 Disaster Management Training and Education Centre for Africa, University of the Free State, Bloemfontein 9301, South Africa

**Keywords:** indigenous crops, smallholder farmers, HFIAS, Poisson regression model, food (in)security

## Abstract

Market participation by smallholder indigenous crop farmers holds significant potential for the alleviation of food insecurity and poverty amongst rural households. Smallholder farmers generally play a vital role in the production and marketing of indigenous crops in South Africa. However, smallholder farmers continue to remain among the food-insecure populations. This is more worrisome for indigenous crop farming households whose produce is far more limited in the market yet may contribute towards improving their food security status and livelihoods. This study analysed the impact of market participation of indigenous crops on the household food security of smallholder farmers in the Limpopo and Mpumalanga provinces of South Africa. A sample size of 209 indigenous crop producers were selected from a population of 1520 smallholder farmers. The study adopted a multi-stage stratified random sampling technique. The data were collected using both qualitative and quantitative research and were analysed using descriptive analysis, Household Food Insecurity Access Scale (HFIAS), and the Poisson regression model with the endogenous treatment model. The household size, marital status, household members living with HIV, and access to extension services were found to be positive and significant in determining household food security, whereas the disability of a household member was significant but negative. While the number of smallholder farmers involved in the production of indigenous crops is still limited, enhanced marketing of indigenous crops may improve the food security status of smallholder farmers. There is still a need for the government to channel its interventions towards the indigenous crop production sector, and this may include the provision of trained extension officers to assist smallholder farmers. Policymakers need to develop policies that support the production and marketing of indigenous crops. More awareness and promotion of indigenous crops are needed to encourage more smallholder farmers to adopt this farming practice. Further study of smallholder indigenous farmers across nine provinces of South Africa should be conducted to obtain deeper and broader insights into the role of these farmers on household food security.

## 1 Introduction

Food insecurity is a global challenge that needs to be met with the development of better food production systems, education, conflict resolution, and other world hunger solutions. The Food and Agriculture Organization (FAO) [1] defined food security as “when all people at all times consume food of sufficient quantity and quality in terms of variety, diversity, nutrient content and safety to meet their dietary needs and food preferences for an active and healthy life, coupled with a sanitary environment, adequate health, education and care”. Although South Africa is regarded as being nationally food secure, food insecurity is still a major challenge for many households and individuals due to a lack of purchasing power [2], and the country faces more serious food security challenges compared to countries with similar income levels such as Malaysia, Brazil, Algeria, and Angola [3]. The country is deemed food secure at a national level as a result of its ability to produce sufficient staple food for all the citizens or the ability to import sufficient food [4]. This, however, contradicts the food security status at the household level, with a huge number of households residing in both rural and urban areas still suffering from food and nutrition insecurity. The number of people vulnerable to food insecurity increased by 2.2 million in 2020 from 11.4 million in 2019 to 13.6 million in 2020 [5]. The percentage of households with food access problems increased from 17.8% in 2019 to 20.6% in 2020, indicating a 2.8% increase. Likewise, the percentage of people with inadequate and severely inadequate access to food also increased from 19.5% to 22.8% during the same period, indicating a 3.3% increase [5]. The deleterious level of food security challenges in the country has been heightened by the COVID-19 situation, which negatively affected a number of people in the country and abroad. The swift assessment of the impact of COVID-19 on food and nutrition security showed that about 48.9% of individuals had moderate to severe food insecurity [6].

The country is implementing a multi-sectoral National Food and Nutrition Security Plan—NFNSP (2018–2023) as part of responding to the food security problems. Several policy decisions and programmes have been implemented to address food and nutrition insecurity over time. For instance, the government has intervened by promoting the development of agricultural projects for potential job creation and household income generation. Mdluli and Dunga [5] reported that nationally, the poverty line has increased from ZAR 585 per person in 2020 to ZAR 624 in April 2021 per person, monthly. This means that a huge number of households in South Africa live beneath the poverty line. Furthermore, nearly 14 million South African households are susceptible to food insecurity as their ability to enter the market and purchase food is diminishing [7]. This is a result of factors such as limited salaries relative to the escalating living costs, extensive unemployment, and dependent ratios for people. Furthermore, poverty is the main food insecurity factor in most developing countries such as Zimbabwe, Zambia, Kenya, South Africa, and Ethiopia, especially in the rural communities in these countries [8–14]. However, growing and selling surplus indigenous crops can be a solution to the underlying issue, thus improving not only the food security situation of the participating farming households but also their nutritional statuses.

These indigenous crops are popular for being highly nutritious and are an excellent source of nutrients such as protein, vitamins A and C, iron, calcium, and dietary fibre [15]. Indigenous crops are defined as plant species that are either genuinely native to a particular region, or which were introduced to that region for long enough to have evolved through natural processes or farmer selection [16]. These vegetables are generally consumed by the rural population and are easily accessible with high nutritional value, yet are underutilised [17]. The underutilisation of these crops is becoming a critical issue in South Africa due to a lack of knowledge about the nutritional and medicinal benefits of these crops. Furthermore, their limited availability also makes it difficult for a massive number of households to obtain and consume them.

A huge number of African rural households have been dependent on these for sustenance. Indigenous crops in the country are mostly produced in rural areas for household consumption with a little surplus that reaches the informal market in some cases through intermediaries. The availability of indigenous crops is limited compared to other food choices, and it is therefore very important to produce them for both consumption and selling. This is because through selling these crops, more smallholder farmers may be encouraged to engage in growing them and thus make them largely available and accessible, even to those who do not grow or produce them through markets. Smallholder agriculture in South Africa has been known as the vehicle through which the objectives of poverty reduction and rural development can be achieved [18]. Smallholder farmers are the ones who are more involved in the market where these crops are being sold. As a result, the availability of indigenous crops improves food and nutrition security due to their nutritional benefits. As indicated, utilising these crops could diversify food choices within the food system and also address household food insecurity and enhance livelihoods, including income generation from indigenous crop sales.

Additionally, the production and consumption of these crops help enhance the Sustainable Development Goals (SDGs), namely, improved nutrition, ending hunger, achieving food security, and promoting sustainable agriculture [19]. The market participation of indigenous crops can help stimulate the income for smallholder farmers so that they can be able to purchase other foods that they cannot produce and be food secure. Selling these crops continuously cannot only allow sustainability among the livelihoods of smallholder farmers but also can reduce their food insecurity because they are diverse on their own [20].

Most of the indigenous crops are still largely collected from the wild and are hardly consumed by households, let alone seen as a potential for income generation for the household. While some studies have been conducted in Africa on a number of agricultural experiments concerning indigenous crops and their economic potential [21–25], South Africa does not have much information on the potential income that could be created through selling indigenous crops and the impact that it would have on reducing food insecurity amongst the market participating farmers in the country. The insufficient context-specific information about the market participation of these crops has resulted in farmers not only being oblivious to their potential contribution to the stability of income and food availability but also how it could contribute to their nutritional status. This has led most households to perceive the trade of indigenous crops as only a temporary activity. Mahlangu [16] reported that there is a market for indigenous crops and therefore it is important to understand the impact of market participation on the household food security of smallholder farmers in South Africa. Therefore, the trading of indigenous crops in South Africa can play a huge role in helping close the food security gap between the national and household levels.

The intervention of smallholder farmers through the provision of these crops cannot only enhance the food security status at the household level but also provide them with sustainable income opportunities to improve their livelihoods as well. Several studies have been conducted on the commercialisation of indigenous crops [26–29]. On the other hand, numerous studies have been conducted on the potential of indigenous crops in improving food security [15,30–32]. There is rather limited information that links both the commercialisation of indigenous crops together with their potential in improving food security. In light of this background, this study is designed to provide insight into the contribution of indigenous crop smallholder farmers’ market participation on household food security.

## 2 Literature Review

Agriculture is a solid contender for empowering economic growth, poverty reduction, and advancing the food security situation in South Africa. It plays a significant role in helping ensure good nutrition and improved livelihoods for poor households [33]. Smallholder farmers play a vital role in ensuring that food is available in the markets. Hawkes [34] pointed out that food production by smallholder farmers is viewed as holding the potential to influence their households’ members’ nutrition, either via direct consumption or indirectly through generating some income which permits them to purchase local food. Dorward and Dangour [35] further added that the food consumption of smallholder farmers together with their nutritional status is normally influenced by what they grow, and therefore own production impacts their diets.

The food security of smallholder farmers is extremely dependent on the income or sales that they obtain from their production. Since the majority of rural smallholder farmers’ production includes indigenous crops, participating in the market sales of these crops could help them a lot because they will gain some income and improve their standard of living. Smallholder farmers are farmers that are located in rural areas and are normally characterised by small plots of land (2 hectares) and poor resources such as capital, farming equipment, labour, and land, while they assume a significant part in poverty alleviation, especially in poor rural areas [36]. This is because they normally plough and grow crops in their backyards. Their production usually includes indigenous crops, simply because they are easy to grow, survive under harsh weather conditions, and do not necessarily require many fertilisers. It is evident from the past literature that smallholder farmers highly benefit from growing indigenous crops in two ways. Firstly, they economically benefit from a rising market, and secondly, through the consumption of these crops, they benefit with regard to their food security level/status because of the nutritional value indigenous crops contain [27]. Thus, this makes their livelihoods highly dependent on farming.

A considerable amount of literature has been published on how market participation of indigenous crops enhances the income of smallholder farmers [23,24,27,37,38]. Muhanji et al. [39] conducted a study on African indigenous vegetable enterprises and market access for small-scale farmers in East Africa. The results showed that smallholder farmers that produced and sold indigenous crops earned a large amount of income compared to the ones that produced exotic vegetables. The study also revealed that as a result of increased consumer demand, even smallholder farmers that only specialised in exotic vegetables shifted their focus and diversified their produce by targeting indigenous crops as their new source of income. All this was due to the income that they all obtained from producing and selling these crops. As a result, their food security status levels increased. These findings were supported by Simon et al. [40], who argued that the Horticultural Innovation Nutrition Research Program project that was undertaken in Zambia and Kenya played a huge role in assisting smallholder farmers to earn high income from commercialising indigenous crops, which in turn positively influenced their food security status levels. This was due to the increased demand for the crops following the training programs (on the production, selling, and preparation methods of indigenous crops) that the project provided, together with the awareness about the nutritional value of these crops. Nonetheless, Nzabakenga et al. [41] found something different in their study. Their results revealed that the smallholder farmers with large pieces of land earned more income due to high productivity levels compared to those with smaller pieces of land. However, even though Ademe et al. [42] discovered that smallholder farmers with larger pieces of land in the highlands of Eastern Ethiopia produced more crops, the market distance hampered their opportunities of obtaining high income. This is because they could not reach the market due to a lack of transport, extension services, and poor infrastructure.

According to Nzabakenga et al. [41], family labour that consists of more women stands a good chance of expanding their agricultural productivity, leading to more income for the households. This is in line with the goal of the Horticultural Innovation Nutrition Research Program project that was undertaken in Zambia and Kenya. Their goal was not only to help provide the smallholder farmers with training and skills on the productivity of indigenous crops but also to ensure that more women participated in the project so that the income will rise [39]. This is because women have been proven to be extremely more productive and hands-on compared to men in the agricultural industry. Moreover, when more vulnerable women are economically empowered, there is a higher number of households with a balanced diet because women are well known for prioritising healthy eating over junk foodstuff. Moreover, a comparative study by Nzabekanga et al. [41] concluded that households with few members tend to generate less income from agriculture as a result of family labour than those with higher members. This leaves the higher income earners to be privileged to diversify their diets.

In addition, through a detailed examination of production diversity, dietary diversity, and consumption seasonality, panel data evidence from Nigeria by Ayenew et al. [43] revealed that an increase in the crop yield together with the high productivity of indigenous crops sold to the markets can lead to a high income of smallholder farmers, resulting in diversity in their diets. Nzabakenga et al. [41] further stated that the purchasing power of many rural households, including smallholder farmers, is extremely determined by agricultural income since agriculture is the backbone of their livelihoods. Therefore, the increase in the production of indigenous crops together with the income can lead to the smallholder farmers being food secure. However, Ochieng et al. [44] postulated that there is a massive number of smallholder farmers with high productions of indigenous crops that still suffer heavy losses of limited income. This is normally due to the lack of market access, storage capacity, market information, and infrastructure [45]. Furthermore, Mayekiso [46] added that the underutilisation of these crops can also be a lack of awareness and nutritional value.

Other studies have been conducted on the impact of income on indigenous crop farmers’ productivity [23,24,47–49]. From a study that was conducted by Nyaruwata [24], it was concluded that the level of income influenced smallholder farmers’ production quantity. The higher the income they received, the higher the quantity they produced on their next production. On other hand, Ogutu et al. [49] discovered that the possession of supermarket contracts directly and proportionally affected the income of smallholder farmers. It was revealed that the smallholder farmers that produced more produce and earned more income were those in possession of supermarket contracts, which in turn helped improved their livelihoods, especially their food security status level. However, Rapsomanikis [47] found that the income received by smallholder farmers was highly influenced by land size. This was due to smallholder farmers with larger pieces of land producing higher quantities than those with smaller land. However, there has been relatively little literature published on the impact of market participation of indigenous crops on household food security of smallholder farmers, especially in South Africa [46,50]; most of the literature is from other African countries [40,43,51]. Opiyo et al. [51] concluded that the marketing of indigenous crops plays a vital role in enhancing the nutritional status of households, especially in rural areas. This view was supported by Mayekiso [46], who also added that lack of knowledge about their nutritional value is the reason why they have been neglected and underutilised.

Therefore, from the existing literature, there is limited research on the impact of the market participation of indigenous crops and households’ food security status. This study will not only add more information and knowledge on that but also combine the two aspects. This is because it is very important that the two are combined since the combination has a high effect on smallholder farmers, especially their food security status level. It will also help bring awareness to the policymakers so that they can intervene by making suitable policies specifically for smallholder farmers experiencing such challenges. All that will lead to an increment in their productivity together with their income, but also can enhance their food security status levels.

## 3 Materials and Methods

### 3.1 Area of Study

This study was conducted in the Provinces of Limpopo and Mpumalanga in South Africa. Both these provinces were considered on the basis of the predominance of small-holder farmers involved in indigenous crop production and are generally populated by smallholder communal farmers whose main livelihood strategies involve participation in agricultural and livestock farming.

Limpopo covers about 125,754 km^2^ of the Northern part of South African area, which can be estimated to 10.2% of the country’s total area. It consists of five districts, namely, Sekhukhune, Capricorn, Mopani, Waterberg, and Vhembe, that are populated with about 5.8 million households [46]. In this province, agriculture is highly prioritised as 89% of the population is not only involved but also dependent on it for their survival. It is most peoples’ occupation. The districts mentioned above were used to conduct the study. The Mpumalanga province on the other hand is situated in the north-eastern part of the country. The province covers 6.5% of South African land area. Moreover, from the 4.04 million population of the province, about 72% are involved in agriculture [46].

Regardless of the fact that both Mpumalanga and Limpopo provinces are occupied by agrarian dependent smallholder farmers, they contribute to the agricultural economy of the country [52,53]. Mpumalanga lies at 665 m above the sea level and consists of temperate and warm weather conditions with about 1000 mm rainfall received per annum [54]. Limpopo, on the other hand, has inconsistent and erratic rainfall distributions. According to Cai et al. [55], this province normally remains dry for three seasons except for the summer season (October–March), where it usually receives about +/ − 500 mm of rainfall per annum. Furthermore, with the climatic conditions that these two provinces experience smallholder farmers manage to grow indigenous crops such as pumpkin, cassava, leafy vegetables, okra, amaranth, and amadumbe.

### 3.2 Data Collection Methods

This study used secondary data. The research data were extracted from a larger food and nutrition baseline assessment study that was conducted in the different provinces in South Africa in order to gain insight into the food and nutrition security situation, as well as the livelihood strategies in the country. Therefore, the data used in this study were conducted by the South African Vulnerability Assessment Committee (SAVAC), led by the Secretariat hosted in the Department of Agriculture, Land Reform, and Rural Development (DALRRD) in 2016 (available at www.drdlr.gov.za accessed on 28 September 2021). The information gathered included but was not limited to demographics; crops (including indigenous crops) produced, consumed, and sold by the farming households; illnesses; livelihoods; and food and nutrition security information. The comprehensive of the 2016 survey and lack of new surveys of the same magnitude in the country that focuses on a household level make these data remain very relevant. No other comprehensive agrarian, food, and nutrition security dataset that is delegated at a household level has been gathered in the country up until now.

The study adopted a quantitative research approach. The multi-stage stratified random sampling technique was used to select households’ representatives’ samples. Multi-stage stratified random sampling is a process that is normally applicable in huge inquiries of geographical data [56]. In this process, each individual has an equal chance of being chosen as a part of the sample [57]. This technique was perfect for the study, not only because it provides detailed and more reliable information about the sample, but also because it saves time and money. There are also the least performance prediction errors with this technique [58]. Similar characteristics such as institutional factors, sales, socio-economic characteristics, household sizes, and outputs were used to divide the farmers in each site. Furthermore, from the two provinces that this study focused on, a total of 1520 respondents were selected, and out of that total number of smallholder farmers, only about 209 were involved in the production of indigenous crops. Therefore, the main focus of this study was 209 smallholder farmers that were involved in the production of indigenous crops only. The focus was not only on their participation in the cultivation of indigenous crops but also on the commercialisation of their produce since their livelihoods solely depend on agriculture for survival. The main intention of this research was to assess the impact that indigenous crops’ market participation of smallholder farmers has on the household food security status level. The smallholder farmers were asked to provide a list of various types of crops that they produced, consumed, and sold to the market. Furthermore, from the list of crops provided by smallholder farmers the indigenous crops were selected (Table 1).

### 3.3 Data Analysis

Statistical analysis of data was performed using Statistical Packages for Social Science (SPSS) (IBM, 2009). In the analyses of data, descriptive statistics, as well as the econometric model, were used. Using the descriptive analysis in this study was beneficial as it quantitatively describes and summarises the sample and observations in a meaningful way. Descriptive statistics are distinguished from inferential statistics, in that descriptive statistics aim to summarise a sample, rather than use the data to learn about the population that the sample of data is thought to represent. According to Maurya et al. [58], “descriptive analysis gives numerical and graphic procedures to summarise a collection of data clearly and understandably”. Furthermore, they stated that “descriptive statistics helps us to simplify large amounts of data sensibly”.

## 4 Econometric Analytical Tools

The HFIAS indicator was used to distinguish between food-secure and food-insecure households, while Poisson regression was used to analyse the determinants of household food insecurity among smallholder farmers. Food access, which dwells on the demand side of food security, has as of late been assigned as one of the significant supporters of food insecurity [59]. In 2006, the Academy for Educational Development the Food and Nutrition Technical Assistance (FANTA) Project was funded by the United States Agency for International Development (USAID) to specifically publish a tool that can be used to measure the access constituent of household food insecurity [60]. The main purposes of the tool development were for it to be applicable, easy to use, and as simple as possible, with only minor adaptations to various sociocultural contexts [60].

However, several different metrics can be used to measure household access to food, namely, the Household Hunger Scale (HHS), the Food Consumption Score (FCS), the Household Dietary Diversity Score (HDDS), household consumption and expenditure surveys, and the Household Food Insecurity Access Score (HFIAS) [61,62]. In this study, we adopted Household Food Insecurity Access Scale (HFIAS).

HFIAS is a legitimate and approved method that is applied in measuring household food access and has been revealed to also measure food insecurity with a satisfactory standard in a few less developed countries [63,64]. It has also been proven to be a success in measuring South African food insecurity through a few studies [4,60,65]. Furthermore, even a developed country such as the United States of America has used the HFIAS in measuring the food insecurity access component [66].

HFIAS was the perfect tool for this study’s research because unlike the Household Dietary Diversity Score, which surveys households on the basis of a 24 h recall period of dietary diversity, it measures an experience of food insecurity that might have occurred within 30 days. Even though the HDDS may be appropriate in promoting a healthy eating pattern through the emphasis on enough consumption of different food groups, it does not necessarily provide any intra-household food distribution information [67]. This is due to the data being collected at the household level. In that way, it provides no information on the utilisation of various food groups or comprehensive dietary diversity by individual people in the household [67]. On the other hand, the information collected from HFIAS was used to assess the frequency of household food insecurity together with the over-time changes amongst the population. This is extremely beneficial in terms of food access-related issues that specifically target, program, monitor, and evaluate the population [68]. Even though it can be said to be a “benefit” bias, it is less expensive and allows for decentralisation [69].

Therefore, the food security status of rural households from the Limpopo and Mpumalanga Provinces of South Africa was assessed using the HFIAS tool. The levels of food security were determined through the creation of the HFIAS score indicator [70]. The HFIAS score continuously measures the experience of food insecurity (access) occurring within the past month. Each household had to indicate whether the experience of food insecurity was as a result of a lack of food or funds to purchase food in the previous month. Then, the score of each household was calculated by tallying the coded prevalence for all the questions concerning food access at the household level, one by one [71]. The standard scoring procedure was 1 (yes) point for occurrence, whereas 0 (no) for non-occurrence. However, besides the occurrence, if the answer of the respondent was “yes” to the occurrence question, then they had to specify the frequency of the occurrence (never, rarely, sometimes, and often) (see Table 2 below). During the tallying of scores from the 9 questions, all the responses were included in the results. The higher scores indicated that the household was food insecure, while the lower scores meant that the household was food secure.

## 5 Theoretical Model

The study used the endogenous Poisson model as the theoretical model.

Following the literature, namely, Terza (1998) and Bratti and Miranda (2010), we regarded the *i*th household that was selected from a random sample *I* = {1 − *n*}. Contingent on a vector of explanatory variables *xi*, a random term *εi*, an endogenous dummy *ci*, and the dependent variable *yi* normally referred to a count were assumed to follow a standard Poisson distribution:

(1) $$f\left(\frac{yi}{\varepsilon }\right)=\frac{\text{exp}\{-\text{exp}(xi\beta +\gamma ci+\varepsilon i)\}\{\text{exp}(xi\beta +\gamma ci+\varepsilon i)\}yi}{yi!}$$

where *β* and *γ* coefficients are to be estimated. Of note, the unobserved and omitted variables including any measurement error were measured by the error term *εi*. Considering a vector explanatory variable *zi* (that might be containing some elements of *xi*), *ci* is characterised by an index process

(2) $$\left\{\begin{array}{l} 1\;if\;zi\alpha +vi>0\\ 0otherwise \end{array}\right\}$$

## 6 Empirical Model

Considering Equation (1) above, the study’s dependent variable (*yi*) was the household food (in)security status level of the smallholder farmers. It followed a Poisson distribution variable since it is a count variable. This was postulated to be determined by the commercialisation of indigenous crops to the market (*ci*) including institutional and demographic variables (*ci*). *Ci* is also affected by some institutional and demographic variables, which, for the intention of clarity, are represented by *zi*. Certainly, both *ci* and *yi* may also be determined by some unobserved variables, such that if the equations for the two variables were estimated separately, the true effect of *ci* could not be measured, including other variables on *yi*.

The models of Terza [71] and Miranda [72] offer a solution the same as that of Heckman’s [73] treatment effect model that amends for problems of bias selectivity in some simultaneous equation models. Furthermore, it should, however, be noted that it is not automatic that there is endogeneity between market access and production. The empirical model to be estimated to measure the effect of the market participation of indigenous crops on the household food security of smallholder farmers is given as follows:

(3) $$\;Commercialization=\alpha_{0}+\alpha_{1}\;AgeFM\;+\alpha_{2}HHSFM+\alpha_{3}\;GenderFM\;+\alpha_{4}EducFM+\alpha_{5}\;MaritalStFM\;$$

(4) $$\;Foodsecurity\;status\;=\beta_{0}+\beta_{1}\;AgeFM\;+\beta_{2}HHSFM+\beta_{3}\;GenderFM\;+\beta_{4}EducFM+\beta_{5}\;MaritalStFM\;$$

Furthermore, it was necessary for the study to create and present dummy variables that were useful in the selected socio-economic knowledge on indigenous crops. The study used the independent variables that are illustrated in Table 3 below.

## 7 Results

Even though the food security status level can be estimated scientifically through scales, such as the Household Insecurity and Access Scale (HFIAS), this paper intended to outline the qualitative aspects of the effect that the production and marketing of indigenous crops had on the household food security of smallholder farmers. The findings are based on comprehensive knowledge that was gathered using questionnaires that were administered to rural farming households.

### 7.1 Demographic and Socioeconomic Characteristics of the Households Involved in the Commercialisation of Indigenous Crops

In this sub-section of the study, we represent the demographic and socioeconomic features of the respondents that participated in the survey. From an analysable sample size of 1520 households, 209 were indigenous crop producers. Out of the smallholder farmers that were involved in the production of indigenous crops (209), only 41 participated in the market through the identified indigenous crops (Table 1). As illustrated from the table, amongst all the other crops, pumpkin was the most produced indigenous crop of them all (4.9%). Leafy vegetables were ranked the second most produced (4.1%). Furthermore, the results revealed that sorghum was the least produced indigenous crop (0.06%). A possible explanation for this could be the fact that sorghum is mainly used as an ingredient compared to other indigenous crops that are practised. Table 4 illustrates these findings.

The different types of indigenous crops that smallholder farmers sold in the market are presented in the table below (Table 5). Surprisingly, the findings revealed unexpected results when leafy vegetables became the most sold indigenous crops, whereas pumpkin was the most produced indigenous crop (Table 4). Furthermore, the results also revealed that some of the indigenous crops were not sold to the market (millet, eggplant, and cowpea). This is due to the limited production of these indigenous crops, inadequate for both consumption and selling. Table 6 illustrates the mean and standard deviations of the market participants and non-market participants.

The access to agricultural assistance appears to protect smallholder farmers from food insecurity. The results revealed that about 48% of the smallholder farmers were market participants, while 43% were non-market participants. The results insinuated that the ones that participated in the market were equipped with the necessary skills and knowledge to improve their production, which leads to improved food security status. On the other hand, regarding a family member that worked on the farm, the results indicated a higher average for the market participants (66%) and lower for the non-market participants (55%). This is because in most cases, smallholder farmers in rural areas mainly rely on family labour for crop production. Having household members that actively participate in agricultural activities plays a huge role in enhancing productivity and reducing vulnerability to hunger since the majority of households rely on agriculture for their livelihoods. Furthermore, as expected, the irrigation type of the market participants showed higher results (58%) than those of the non-market participants (36%). The results proved that irrigation is important in ensuring that households attained food security. Moreover, this implied that the market participants did not only rely on the rainfall for their crop yields but also had other means to irrigate their crops which assisted in promoting their crop production. Irrigation plays a significant role in reducing the risks of crop failure [74].

### 7.2 The Food Security Situation among the Surveyed (Farming) Households

**Figure 1:**
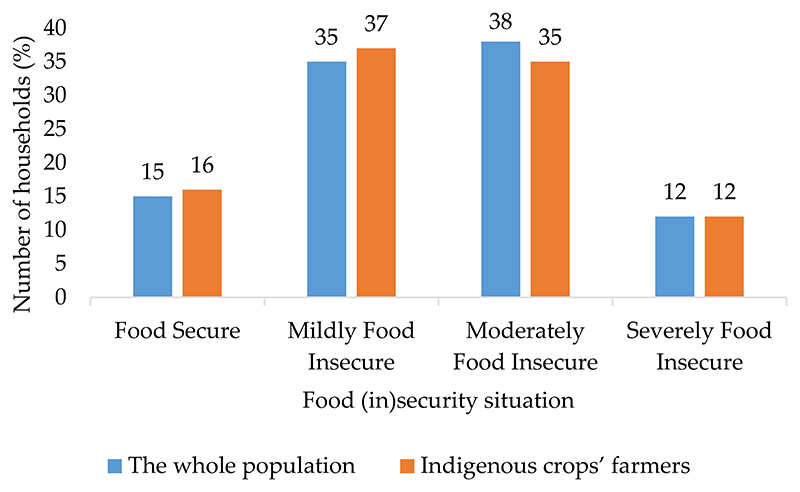
The food (in)security situation of the whole sampled population and indigenous crop farmers.

Figure 1 shows the occurrence of food insecurity among the whole sampled population and indigenous crops farmers. The results revealed that out of the total sample size, 85% of the sampled households were food insecure and only 15% were food secure, indicating that the majority of the households were experiencing difficulties when it comes to food access while only a few percentages had no difficulties in accessing food. Figure 1 showed that when food (in)security is categorised into four as determined by the HFIAS tool, the majority of the households were moderately food insecure, with 38% of the households being found in this category. This was followed by mildly food-insecure households, with 35% of the household being in this category. More worrying is that 12% of the households were found to be severely food insecure, indicating serious problems relating to access to food in those surveyed households.

## 8 Source: Author’s Own Analysis

Analysis of the food security situation amongst the indigenous crop farmers revealed that the majority of the farmers were food insecure, with only 16% of the indigenous farmers found to be food secure (Figure 1). When categorised, the results indicated that most of the indigenous crops farmers were mildly food insecure, with 37% of the households being in this category. The mildly food-secure households were struggling with the availability of limited diversity and quality of food intake. Conversely, 35% of the indigenous crop farmers were found to be moderately food insecure, which could be associated mainly with the need to consume the kinds of quality food or the challenge of consuming less preferred food. It was also revealed that about 12% of these farmers were severely food insecure, demonstrating that some of them experienced difficulties in accessing food.

The results in Table 7 highlight the impact of market participation of indigenous crops on household food security of the surveyed smallholder farmers. The results revealed that the household size, marital status of a family member, HIV status of a family member, and access to extension services were all significant with positive coefficients. Furthermore, despite disability, if a member receives a grant for tjeing disabled, this was significant but had an unexpected negative coefficient.

The results indicated that the household size had a positive impact on the market participation of the indigenous crop household food security status level of smallholder farmers and was statistically significant at the 1% level. The marital status of a family member also showed a positive impact, with a 10% level of statistical significance. The results of a member that received a disability grant indicated a negative impact but was statistically significant at the 10% level. HIV infection of a member of the household revealed a positive impact on the market participation of indigenous crops on the household food security status level of smallholder farmers and was statistically significant at the 10% level. Access to extension services also indicated a positive impact with a 1% level of statistical significance.

## 9 Discussion

This study investigated the impact that producing and selling indigenous crops in the market had on the household food security of smallholder farmers. The study’s motivation was to obtain information that could be useful and documented but also used to promote the consumption of indigenous crops. Of note, the results showed that partaking in the market of indigenous crops plays a significant role in the household food security of smallholder farmers.

The results indicated that household size had a positive impact of market participation of indigenous crops on the household food security status level of smallholder farmers and was statistically significant at the 1% level. The marital status of a family member also showed a positive impact with a 10% level of statistical significance. The results of a member that received a disability grant indicated a negative impact but statistically significant at level 10%. HIV infection of a member of the household revealed a positive impact of market participation of indigenous crops on the household food security status level of smallholder farmers that was statistically significant at the 10% level. Access to extension services also indicated a positive impact with a 1% level of statistical significance.

The results of this study also indicated that the household size was positive and significant, suggesting that the size had a higher probability of influencing the study’s participants’ food security status. Normally, smallholder farmers in rural areas mainly depend on family labour for the production of indigenous crops. Therefore, households with more members can overcome labour by dividing the agricultural activities and indigenous crops to be grown among themselves. Moreover, more members mean that they can overcome financial constraints (from non-farm income activities), which then promotes the affordability of their diverse food choices that provides their bodies with nutritional benefits. Thus, this study’s results indicated that there is more diverse produce of indigenous crops enough for both consumption and surplus to be sold to the market. This allowed the households to buy and consume all their nutritious diets, and hence food security. This was contrary to the findings of Omotayo and Aremu [75], where the size of the household had a negative coefficient but was significant. Omotayo and Aremu [75] concluded that the lesser the number of members in the household, the lesser the demand for food. Moreover, Rubhara [76–78] found that household size positively influenced household food security and was significant. However, the justification of Rubhara [76] was that the increase in household size will inevitably increase the demand for food, and the available food may not be adequate to satisfy the increased demand.

In addition, from the results, it was also revealed that the marital status of the household head increased the chances of being food secure. The marital status coefficient was positive and significant. The possible explanation for these results is that married households made informed joint decisions about the production of indigenous crops. Also important was the diversity of the crops produced and sold to the market. Therefore, this provided them with income enough to purchase a variety of food for their diets. Similar findings were reported by [50,79,80]. Their results revealed that the marital status of the household head and food security had a positive and significant relationship. The justification from these studies was that joint relationships strive to provide households with diverse nutritious food, enhancing the chances of being food secure. However, these findings seem to contradict those that were found by [75,81,82], which had a negative impact and were not significant.

The results showed that the disability grant received by a household member had a negative coefficient but was significant. The negative coefficient indicated that the dependence on disability grants had a negative impact on households’ decisions to grow indigenous crops. Initially, the grants were meant for promoting livelihoods and improving the economic activities of the households by easing their financial constraints. Furthermore, it was for their living standards to improve. However, a possible explanation for the results of this study could be that households are no longer interested in growing indigenous crops but completely dependent on the grant for their livelihoods. These results were consistent with the results of earlier studies [83,84] that found that households no longer cultivate land because they receive a social grant. However, Sinyolo et al. [85] found contrasting results. They found that social grant was not significant and did not affect the households’ food security nor relax their financial constraints. Furthermore, Zaca et al. [86] found a positive relationship between households that received a grant and their food security status level.

Furthermore, the human immunodeficiency virus (HIV) variable revealed interesting results. A household with HIV-positive members positively influenced household food security and significance. As a result of people living with HIV having to maintain a balanced healthy diet, the results implied that in such households, they diversified their diets through being involved in the production of indigenous crops. Furthermore, this also indicated that the households cultivated a greater diversity of indigenous crops enough for consumption, then selling the surplus to the market to generate additional income. This, however, appears to protect households from food insecurity. This is in line with a study that was conducted by [87], which discovered that HIV-infected households prioritised growing vegetables and consumed food choices that would provide them with the necessary requirements of the body. However, Ladzani [88] and Makwangudze [89] as well as Thornton [90] found contrasting results and postulated that HIV jeopardises the livelihoods of the households because it minimises their working capacity and productivity, and hence most of them are food insecure.

As expected, the study found that the access to extension services were significant and positively influenced the food security of smallholder farmers. A possible explanation for this could be the fact that such services advise and encourage smallholder farmers to be involved in the production of indigenous crops and provide them with information on new technologies and market access. Furthermore, extension services not only provide ways to increase the productivity level of farmers but also the application of relevant knowledge in response to diseases and pests. Therefore, the increment of productivity means more produce being sold to the market and more income. This then leads to them buying diverse diets of their food choices. The results were substantiated by earlier findings by [91–94]. Their findings reported that extension services also help provide smallholder farmers with agricultural skills, intelligence, and knowledge to facilitate market access and trade. Justus et al. [44] revealed that the extension services provide smallholder farmers with the inability of consuming nutritious diverse diets.

## 10 Conclusions and Recommendation

Even though smallholder farmers play a crucial role in contributing to food security in South Africa, especially at the household level, they account for a considerable portion of the food-insecure individuals in the country. The production and sale of indigenous crops can help reduce the problem of food insecurity among smallholder farmers. This study was set out to assess the impact of market participation of indigenous crops on the household food security of smallholder farmers. The household size matters and can provide the required labour to partake in the production of indigenous crops, which the household may sell to gain income to purchase other foods that they cannot produce. The marital status of the household head and access to extension services positively influence the food security status of the farmers involved in the production and sale of indigenous crops. Reliance on disability grants negatively affected the food security status, and therefore there is a need to decrease dependency on disability grants. This kind of study requires some time to be completed. Thus, for further research, time should be prioritised. There is a need to create awareness and also conduct training on the need to diversify livelihoods and income sources. Training is needed to help empower rural farming households about the importance of consuming and selling indigenous crops, particularly because these crops have nutritional benefits and therefore can help reduce food and nutrition security problems in the country. Extension support through the provision of more extension officers competent in the area of indigenous crops is needed. Policies that support the production and marketing of indigenous crops need to be developed. The study investigated the impact of market of participation on the food security of indigenous crop smallholder farmers in two provinces. The study was not originally conducted for indigenous crop farmers; hence, the small sample size of 209. Further research should be conducted in all nine provinces of South Africa.

## Figures and Tables

**Table 1:** Indigenous crops grown by smallholder farmers in the Limpopo and Mpumalanga provinces of South Africa.

Indigenous Crops	Scientific Names
Amadumbe	*Colocasia esculenta*
Bambara groundnut	*Vigna subterranea*
Cassava	*Manihot esculenta*
Cowpea	*Vigna unguiculata*
Eggplant	*Solanum melongena*
Leafy vegetables	
Millet	*Panicum miliaceum*
Okra	*Abelmoschus esculentus*
Pumpkin	*Cucurbita*
Sorghum	*Sorghum bicolor*

Source: authors’ own analysis.

**Table 2:** Frequency of occurrence of food access difficulty among the surveyed households in Limpopo and Mpumalanga.

Do You or Your Household Members Have the Following Problems with Ensuring Food Security Due to Financial Problems/Lack of Resources:	Last 30 Days			
	Never	Rarely (1–2 Times)	Sometimes (3–10 Times)	Often (More than 10 Times)
Worry about not having enough food	378	488	504	150
Do not eat your kinds of preferred food	216	511	548	182
Limit the diversity/quality of meals	260	517	240	283
Consume some foods that you really did not want to eat	263	513	535	208
Limit eaten food portions	401	516	467	136
Limit the number of meals	452	482	431	153
No food to eat of any kind in your household	866	331	254	69
Go to sleep at night hungry	1132	209	109	70
Go a whole day and night without eating anything	1227	147	94	52

Source: authors’ own analysis.

**Table 3:** Estimated factors that affect the decision for market participation.

Variable Name	Variable Definition	Variable Type and Measurement	Estimated Effect of Market Determinants on Food Security	Results Received by Sinyolo et al. (2016)
Age	Age of the household head	In years (continuous)	±	−
Gender	Gender of the household head	Dummy (1 = male, 0 = female)	+	−
Marital status	Marital status of the household head	Marital status (1 = married, 0 = single)		+
Household size	Number of members of the household	Size of household (continuous)	−	−
Educational attainment	Education level of the household head	Education level (continuous)	+	−
Livestock	Ownership of livestock	Dummy (1 = yes, 0 = no)	±	−
Distance	Distance to the market	In kilometers (continuous)	−	−
Credit access	Access to credit	Dummy (1 = yes, 0 = no)	+	+
Extension services	Access to extension service	Dummy (1 = yes, 0 = no)	+	−

**Notes**: ± indicates whether the hypothesised effect will be positive or negative; + indicates a positive estimated effect, and − indicates the negative estimated effect. Source: authors’ own analysis.

**Table 4:** The distribution of indigenous crops.

	Indigenous Crop Producers in the Mpumalanga and Limpopo Province	
Indigenous Crops	Frequency	Percentage
Sorghum	1	0.06
Pumpkin	75	4.9
Okra	4	0.26
Millet	3	0.20
Leafy vegetables	63	4.1
Eggplant	4	0.26
Cowpea	4	0.26
Cassava	4	0.26
Bambara groundnut	15	1
Amadumbe	36	2.4

Source: authors’ own analysis.

**Table 5:** Number of smallholder farmers who sold the identified different types of indigenous crops in the market.

	Smallholder Farmers Who Sold Indigenous Crops	
Indigenous Crops	Frequency	Percentage
Sorghum	1	0.06
Pumpkin	8	0.53
Okra	2	0.13
Millet	0	0
Leafy vegetables	15	1
Eggplant	0	0
Cowpea	0	0
Cassava	2	0.13
Bambara groundnut	3	0.20
Amadumbe	10	0.66

Source: authors’ own analysis.

**Table 6:** Demographic characteristics of smallholder farmers in Limpopo and Mpumalanga provinces, South Africa.

	Market Participants		Non-Market Participants		Pooled	
Variables	Mean	Standard Deviation (SD)	Mean	Standard Deviation (SD)	Mean	Standard Deviation (SD)
Gender of the household head	0.556	0.112	0.534	0.100	1.27	0.45
Household age	46.679	13.342	45.045	12.666	49.12	11.89
Marital status of the household head	0.476	0.356	0.468	0.344	4.21	2.44
Household size	4.776	1.223	3.889	1.012	4.93	2.71
Educational level of the household head	6.778	3.048	5.345	2.345	33.58	40.30
Ownership of livestock	0.567	0.357	0.690	0.327	1.77	0.42
Distance to the market	0.475	0.356	0.474	0.245	1.86	1.82
Access to market information	0.574	0.785	0.475	0.676	1.94	0.24
Access to agricultural assistance	0.476	0.345	0.427	0.367	1.92	0.27
A family member with HIV	0.434	0.432	0.378	0.421	0.47	0.79
Family member worked on farm	0.657	0.557	0.554	0.447	0.98	0.76
Social grant	0.473	0.367	0.488	0.455	1.99	0.73
Irrigation type	0.576	0.234	0.356	0.345	1.52	0.50
Total output of crops (kg)	3557.6	2356.8	2567.2	2345.7	3556.22	88,187.067

Source: authors’ own analysis.

**Table 7:** Impact of market participation of indigenous crops on household food security of smallholder farmers: Poisson with endogenous treatment model.

HFIAS	Coef	Std. Err.	*p*-Value
Age of the household head	0	0	0.616
Household size	0.014	0.003	0.000 ***
Gender of the household head	−0.003	0.033	0.918
If the household head resides in the home	−0.125	0.08	0.12
Education of the household head	0.148	0.091	0.102
Marital status of the household head	0.321	0.164	0.051 *
Access to agricultural assistance	−0.026	0.024	0.274
Ownership of livestock	−0.121	0.113	0.285
If member worked for a wage salary	−0.013	0.064	0.837
WEATHINDEX	−0.019	0.039	0.632
HH received advice from government	0.029	0.028	0.296
Disability if a member receives grant	−0.312	0.169	0.065 *
A family member with HIV	0.151	0.08	0.059 *
1.Commercialization	0.393	0.036	0.000 ***
_cons	2.474	0.151	0.000 ***
Commercialization			
Access to extension	0.137	0.028	0.000 ***
_cons	0.243	0.034	0.000 ***
/athrho	−2.845	0.238	0.000 ***
/lnsigma	−1.591	0.073	0.000 ***
rho	−0.993		
sigma	0.204		
Wald test of indep. eqns. (rho = 0): chi2(1)	142.71		
Prob > chi^2^	0.0000 ***		

Note: ***, and * indicate significance at 1% and 10% levels, respectively. Source: authors’ own analysis.

## Data Availability

Restriction apply to the availability of these data. Data was obtained from the Department of Agriculture, Land Reform, and Rural Development (DALRRD) and are available from South African Vulnerability Assessment Committee (SAVAC) secretariat with the permission of Department of Agriculture, Land Reform, and Rural Development (DALRRD).
